# The Influence of SARS-CoV-2 Variants on National Case-Fatality Rates: Correlation and Validation Study

**DOI:** 10.2196/32935

**Published:** 2022-05-24

**Authors:** William A Barletta

**Affiliations:** 1 Department of Physics Massachusetts Institute of Technology Cambridge, MA United States

**Keywords:** SARS-CoV-2, COVID-19, variants of concern, case-fatality rates, virulence, vaccine effectiveness, correlation study

## Abstract

**Background:**

In 2021, new variants of the SARS-CoV-2 virus appeared with increased transmissibility and virulence as compared with the original wild variant. The first variants of concern (VoCs), Alpha (B1.1.7) and Gamma (P.1), first appeared in the United Kingdom and Brazil, respectively. The Delta (B.1.617.2) variant, seen in India in October 2020, dominated COVID-19 infections across all regions through the second half of 2021.

**Objective:**

This research explores the degree to which SARS-CoV-2 VoCs generate waves of fluctuations in case-fatality rates (CFRs) across countries in several regions, increase the risk of mortality to persons with certain comorbidities, and decrease the risk of mortality as the percentage of fully vaccinated populations increases.

**Methods:**

This analysis introduces a measure of the temporal dynamics of COVID-19 infections in the form of a proxy CFR (pCFR), which can be compared among countries. It uses economic and demographic data reported by the World Bank and International Monetary Fund, plus publicly available epidemiological and medical statistics reported to the relevant national and international public health authorities. From these ecological data, pandemic average and daily COVID-19 CFRs and their correlations with potential cofactors were computed for 2021, a year dominated by the spread of World Health Organization–designated VoCs. The study does not investigate disease pathology; rather, it compares the daily case rates and pCFRs to reveal underlying contributing factors that vary from country to country and region to region.

**Results:**

The in-depth global regression analysis of cofactors found that the strongest single correlation with COVID-19 fatality was 0.36 (SD 0.02) with *P*<.001 for chronic kidney disease. No other single physiological cofactors display positive correlations exceeding 0.26 (SD 0.26), with *P*=.008 (asthma) and *P*=.01 (coronary disease). The study confirms that the pCFR is a valuable metric for tracking waves of infection due to different VoCs within countries.

**Conclusions:**

The influence of social, economic, and medical cofactors on the CFR due to VoCs remains qualitatively similar, albeit strengthened, to the levels found for the wild strain. The strong regional variations of the influence of all cofactors observed for the wild strain persists in infections for all VoCs with very strong correlation coefficients seen in the Middle East for asthma (0.76), coronary heart disease (0.60), lung disease (0.70), and chronic kidney disease (0.52). Strong regional variations emphasize the influence on COVID-19 mortality due to regional differences in national economics, patterns of health care policies, and variations in cultural practices and environment. The pCFR-based analysis reveals clear patterns of the spread of VoCs across regions, but there is little evidence for the spread of the Lambda and Mu (B.1.621) variants of interest outside of South America.

## Introduction

### Background

**Figure 1:**
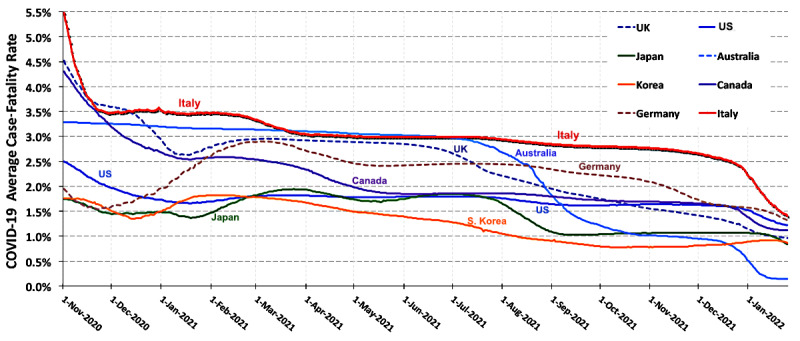
The pandemic's average case-fatality rate for the period during which other variants of concern became widespread.

The period from November 2020 to the end of 2021 is characterized by the rapid spread of several “variants of concern” (VoCs) of the SARS-CoV-2 virus [1-3] across most highly populated nations with both increased levels of transmissibility and virulence. In January 2021, the Alpha (B.1.1.7) variant [4] began its spread from the United Kingdom across Europe. The Gamma (P.1) variant in Brazil [5], first seen in mid-November 2020, began to dominate infections in South America during 2021. During mid-2021, the Delta (B.1.617.2) variant [6,7], first seen in India, became the dominant source of COVID-19 in North America, Asia, and Europe. Figure 1 shows the pandemic’s average case-fatality rate (CFR) for the period from November 1, 2020, through January 2022, when multiple VoCs, in addition to Alpha (B.1.1.7), were widespread.

The rationale for this investigation is to explore the degree to which new VoCs have increased the susceptibility for severe consequences to COVID-19 for persons with common comorbidities and to examine how national vaccination policies may have affected the severity of health outcomes of variant-induced infections. With respect to the influence of comorbidities on COVID-19 outcomes, other researchers have pointed out the shortcomings of the usual warnings by the US Centers for Disease Control and Prevention (CDC) [8,9].

A recent study of four unique endotypes of patients hospitalized with COVID-19 infections found that high-level comorbidities did not associate with poor outcome endotypes [10,11]. Previously, a study on the proximate and underlying causes of death as determined by the autopsy of 26 hospitalized patients found that death was “directly related to COVID-19 in the majority of patients.” Pre-existing health conditions had only contributory implications, and death was not an immediate result of those comorbidities [12]. From the outset of the pandemic, patient age has been a frequently cited cofactor contributing to the severity of COVID-19. Although Alpert et al [13] described a clinical definition for immune age, its application here would require an extensive set of patient data not available for a country-by-country study of national populations.

An earlier study by Barletta [14] examined correlations of COVID-19 fatalities due to the wild strain in 2020, with 15 medical cofactors and eight socioeconomic cofactors (listed in Multimedia Appendix 1). The statistical bases of that study were national statistics of SARS-CoV-2 with respect to the original strain of the virus through December 2020. Since that time, the number of reported cases of COVID-19 has increased from 82.9 million to 288.3 million as of December 31, 2021. Over the same period, the number of deaths increased from 1.81 million to 5.44 million. During 2021, more than 4.56 billion people received at least one dose of an anti–COVID-19 vaccine, and more than 3.82 billion are considered fully vaccinated. COVID-19–related data for all countries was taken from Our World in Data (OWID) [15].

### Specific Objectives

The principal objectives in this study are to establish a valid proxy national CFR and to assess its daily fluctuations, to investigate on a global and regional basis the correlation between average national pCFRs and potential cofactors/comorbidities, and to describe by region the correlation between proxy national CFRs of country pairs.

## Methods

### Temporal Dynamics

The analysis of this study starts with an examination of the temporal behavior of the pandemic’s average CFR as shown in Figure 1. Previously Ghani et al [16] suggested a time-sensitive metric for novel infectious diseases in the context of severe acute respiratory syndrome, explicitly considering recoveries from reported cases. However, that study did not account for the time delay between the first report of infection and the date of the subsequent outcome; however, that consideration is unimportant for a metric averaged over the duration of a lengthy persistent pandemic such as that produced by SARS-CoV-2. The curve for Italy exemplifies how high values of the average CFR in early 2020 reduced the sensitivity of this metric as convincing evidence of waves of increased virulence of the VoCs that spread in 2021. Figure 1 displays a long period during which the CFR in the United Kingdom increased, probably due to the B.1.1.7 variant, and then fell as Britain’s vigorous testing, vaccination, and infection characterization programs took hold. Similarly, the increase in the German CFR in early 2021 is likely driven by the B.1.1.7 variant; however, laboratory data characterizing the variant of the infections in Germany were not available to substantiate that hypothesis. The fluctuations in the CFR in Figure 1 for Australia, Japan, Korea, and the United States are not readily explainable from the pandemic averaged data.

Although the pandemic averaged data are suggestive, they are far from dispositive. Further analysis requires introducing a proxy measure of the CFR, more sensitive to temporal variations in the virulence of the dominant variant but far less sensitive to systematic irregularities in the timing of government reports of fatalities ascribed to COVID-19.

Fourier analysis of the time series of daily reports of new COVID-19 infections displays an unambiguous isolated peak in the frequency spectrum at 1 per week. This manifest systematic irregularity in the reported data (with far fewer cases and deaths on weekends) plus the inherent statistical noise in the data both justify introducing a proxy for the daily CFR. Using an appropriate proxy rate, one can then explore whether the daily CFR in several countries shows evidence of more (or less) virulent variants taking hold or whether robust programs of COVID-19 testing plus vaccination, including boosters, decrease the mortality rate of the disease.

### Study Design

To explore correlations and temporal variations of the influences of VoCs, this study introduces a credible proxy for daily CFRs, which will be sensitive to the extent of the spread of a variant throughout a country. The definition of a suitable proxy CFR (pCFR) and the subsequent analysis and validation of its temporal distribution on a country-by-country basis are presented in the Results section. To evaluate changes in the susceptibility to cofactors, this study follows the methodology of Barletta [14], in which the input data are based on national epidemiological statistics for COVID-19 and potential cofactors as reported to the relevant national and international authorities and tabulated by OWID [15] and the US CDC [8].

### Data Sources and Setting

For consistency with the previous analysis [14], this study analyzes the same sample of 99 countries as listed in Table A.1 of Multimedia Appendix 1. These countries from the Americas, Asia, Europe, and the Middle East had been selected as representative of those having the most reported COVID-19 infections during mid-2020; their population is 5.5 billion persons. At present, the countries omitted represent less than 3% of the world’s reported COVID-19 cases. Although using sex-disaggregated data would have been preferable, a suitable self-consistent data set, disaggregated by sex and ethnicity, has not been reported or is not publicly available for many of the countries included in the analysis. The grouping of countries by region serves as a quasi-proxy for ethnicity data. The focus on the time series of the pCFR and daily infections allows one to observe and, if necessary, adjust for seasonal variations.

### Susceptibility With Respect to Comorbidities

A further question is whether national populations infected with the VoCs display different susceptibility with respect to comorbidities and economic cofactors than they did to the wild variant of the virus. To answer this question, one can analyze correlations of potential contributing cofactors during the period from January 1 through December 2021 over the same set of countries studied previously by Barletta [14].

The potential cofactors that are evaluated with respect to their correlation with fatalities in COVID-19 infection are grouped into three main categories:

1. Physiological characteristics: age and BMI
2. Cofactors: obesity, hypertension, inflammatory heart disease, coronary disease, asthmas, lung disease, lung cancer, susceptibility to influenza-induced pneumonia, chronic kidney disease, leukemia, COVID-19 testing, and reported COVID-19 cases per million persons
3. Socioeconomic and political factors: adjusted gross domestic product (GDP), national health care expenditures, World Health Organization (WHO) health care index, malnutrition mortality, hospital beds per 1000 persons, percentage of population fully vaccinated, number of persons per household, percentage population in urban centers, and percentage of population in slums

Data related to COVID-19 infections are those tabulated daily in OWID [15]. The relevant data regarding comorbidities, as reported to the WHO, can be found in World Health Rankings [17].

### Analysis Methodology

The statistical data analysis used in this paper proceeds in the following order:

1. Plot the pandemic averaged CFR against all individual potential cofactors on a region-by-region basis to explore potential relationships between the CFR and potential cofactors (examples are shown in Multimedia Appendix 1, Section C)
2. If plots of the CFR against potential cofactors display no strong evidence of nonlinear effects when fit with trial trend lines, compute the linear correlation of the pandemic averaged CFR and potential cofactors using the Pearson “product moment correlation” (specific examples with linear fits per region appear in Multimedia Appendix 1, Figures C.1, C.2., and C.4b)
3. Compute the linear correlations between the average CFR for 2021 and all potential cofactors for country pairs both globally and region by region using the data analysis package of Excel version 16.43 (Microsoft Corporation)
4. Absent evidence of significant nonlinear effects as determined in step 2, perform a detailed linear regression analysis of all 24 potential cofactors with the set of national pCFR values, using the standard data analysis package of Excel version 16.43
5. To compare results of correlations of national data within regions, consider country pairs for which the spread of a VoC is likely due either to extremely high transmissibility or due to significant travel of persons across national borders
6. To compare experience in several countries, compare and contrast the time series of daily CFRs

Unfortunately, the raw reported data are noisy, as they are subject to uneven reporting of both new cases and deaths attributed to COVID-19 as well as to inherent statistical fluctuations in the daily data. Moreover, computing the daily CFR on day N as defined in [15]:


Trial daily CFR (N) = 〈Deaths (N)〉 / 〈Cases (N)〉 **(1)**

in which the brackets,〈 〉, denote a 7-day rolling average, yields misleading values for the CFR on day N because the deaths on that day had to be caused by COVID-19 infections that began generally 2 to 3 weeks earlier.

To mitigate these deficiencies in the data, one introduces a plausible proxy, pCFR, for the apparent daily case-fatality ratio. The pCFR is a retrospective diagnostic that compares the deaths on a given day against the average number of new cases during a period from 14 days to 14 + M days prior to that given day. The model overlays those data with a rolling 14-day average of the results to suggest the actual temporal trends in the virulence of SARS-CoV-2 infections. Noting that substantial consequences of infection often appeared within a 7-day period from 14 to 21 days (the range of M) after the reported symptomatic infection, one can define the proxy pCFR by equation 2.



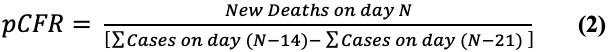



The time series of the trial CFR of equation 1 correlates only moderately well with that of the pCFR. Sample calculations for the United States and the United Kingdom yield correlation coefficients of 0.74 and 0.65, respectively, suggesting that the statistics of SARS-CoV-2 contagion and COVID-19 fatalities do not change rapidly over a 2- to 3-week timescale. To examine the sensitivity of the pCFR to the averaging period of the number of cases that influence the number of deaths on day N, one can change N-21 to N-28 in the denominator of equation 2. The correlation of the resulting two time series ranges from 0.92 to 0.98; hence, the results of the analysis depend only weakly on the period over which the pCFR is computed. To reduce further artificial variations caused by irregularities in reporting, this study uses the smoothed daily deaths computed by OWID [15].

A second trial proxy might be the ratio on day N:



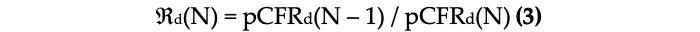



where d represents the rolling average over d days in equation 3. Yet another alternative might be the derivative 
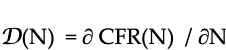
. Unfortunately, like most differential measures, both 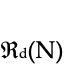 and 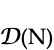 are extremely noisy functions that obscure even strong variations in the CFR. An example of 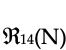 is shown in Multimedia Appendix 1, Section B for the case of the United Kingdom.

## Results

### Temporal Dynamics

The correlation analysis of the previous study [14] of average CFRs of COVID-19 with potential comorbidities and societal cofactors relied on data reported to governmental authorities from March 2020 through October 2020. During that period, the WHO had not yet designated any VoCs [1], although cases subsequently attributed to the B.1.1.7 (Alpha) and B.1.351 (Beta) strains dated from mid-October 2021 and mid-May 2021, respectively [5]. Consequently, the correlations of Barletta [14] were all attributed to the wild strain of the virus even though some cases—especially those in South Africa—may have been more properly attributable to the B.1.351 variant. By mid-December 2020, the WHO had designated both the B.1.1.7 strain and the B.1.351 strain from South Africa as VoCs.

With many European nations included in the data set of this study, the initial date of November 1, 2020, was set for the analysis of the effect of VoCs on virulence and on transmissibility and spread of the disease, and the influence of cofactors in the presence of new VoCs.

### Effects of COVID-19 Vaccines

The level of vaccine-induced immunity in respective populations is a potential cofactor in tracking the dynamics of SARS-CoV-2. Doubtless, one may expect the national reports of the number of new COVID-19 infections, the CFRs, and the reproduction rate of the SARS-CoV-2 virus to be influenced by the degree to which a nation’s population is fully immunized by vaccines. Therefore, those statistics have been analyzed versus the percentage of total population fully vaccinated. A table of examples is given in Multimedia Appendix 1, Section C.

A limitation of the pCFR, likely shared by other daily measures of fatality rates, is that it is most subject to large fluctuations when the COVID-19 daily case rate—and therefore the death rate—is small. That situation often happens when the fraction of total population fully vaccinated exceeds 40% to 50%, especially when other prophylactic measures contribute strongly to driving the reproduction rate, R_o_, to less than 1. The positive aspect of this sensitivity of the pCFR when case numbers are small is that highly variable trends in the pCFR can spot surges of cases in clusters of unvaccinated persons or in less than vigilant groups.

**Figure 2:**
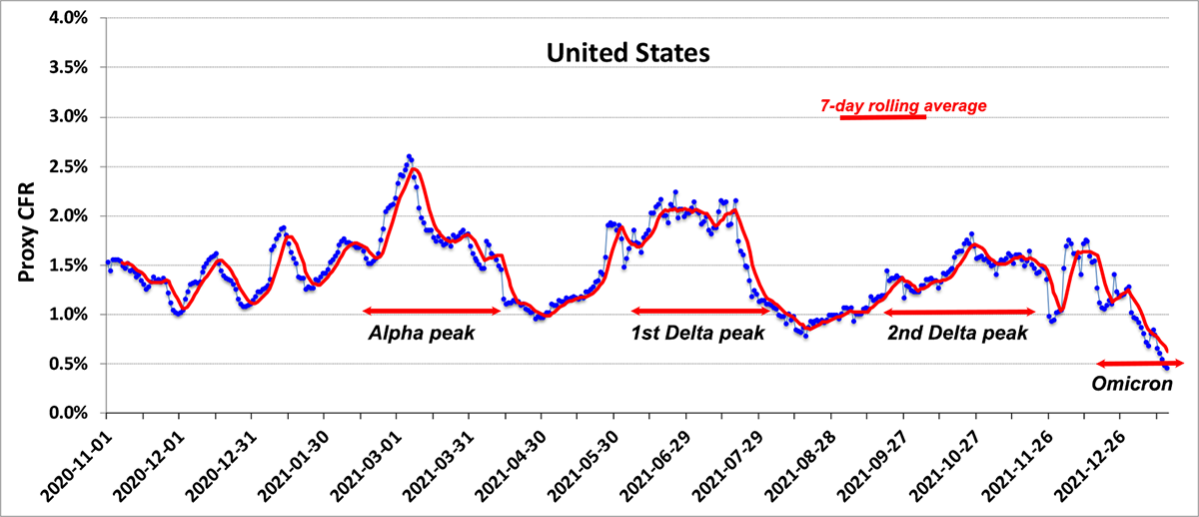
Daily proxy CFR values and waves of infection in the United States. CFR: case-fatality rate.

Figure 2, which displays the time sequence of the pCFR for the United States, shows clear evidence that was not readily visible in the pandemic averaged CFR for the differences in rates of mortality in February and March 2021 due to the B.1.1.7 variant that appeared in the United States in January 2021 [4], before less than 0.6% of the population had received vaccinations. The CDC [4] had predicted a peaking of the number of infections due to B.1.1.7 in March 2021; that surge in cases likely accounts for the increase in the pCFR seen in March 2021.

**Figure 3:**
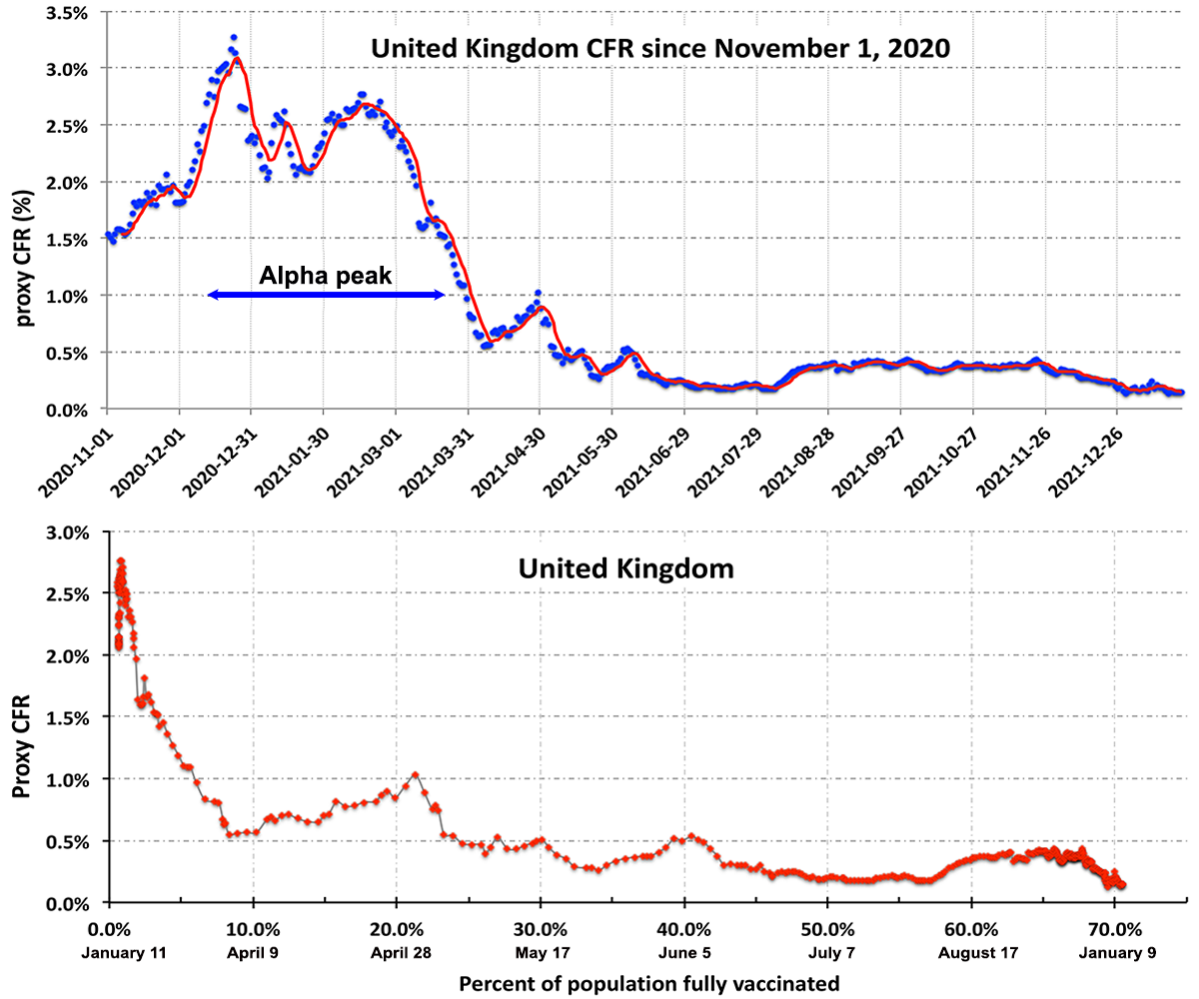
Upper panel: Daily proxy CFR in the United Kingdom since November 2020. Months are in cyan and magenta. The red line is a 7-day rolling average. Lower panel: Proxy CFR for the United Kingdom vs percentage of the population fully vaccinated. CFR: case-fatality rate.

In contrast, the United Kingdom reported infections from the B.1.1.7 variant in mid-December. The variant spread quickly, raising the pCFR to ~3% before any significant fraction of the UK population could be vaccinated. Figure 3A shows the marked increase in the pCFR in late December and January. By March 2021, roughly 30% of the total UK population had received their first dose of the vaccine, and by the end of April, over 80% of the population ≥59 years of age had received their first dose [18]. The pCFR began to decrease steadily in March 2021.

With a vigorous program of both testing (at twice the rate of the United States) for SARS-CoV-2 infection plus full immunization exceeding 68% of the total UK population by November 2021, the pCFR in the United Kingdom has fallen below 0.5%, comparable to other countries in Europe and commensurate with levels sometimes associated with seasonal influenza. The effects of the vaccination program on the pCFR are clear in a plot of the pCFR versus the percentage of the total population fully vaccinated [15] (Figure 3b). The strong prolonged increase in the pCFR during the time of B.1.1.7 dominance is consistent with clinical reports by Twohig et al [7] of increased mortality due to the B.1.1.7 strain. Unfortunately, complete sex-disaggregated and ethnically disaggregated data sets are not available for full comparison with the results of Twohig et al [7].

**Figure 4:**
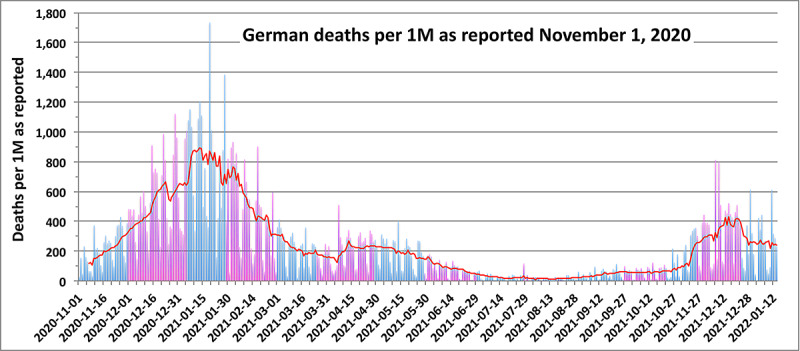
Daily deaths as reported for Germany since November 2020. Months are in light and dark bands. The red line is a 7-day rolling average.

Figure 4 illustrates the case of Germany—intermediate between that of the United Kingdom and the United States. The slower spread of the B.1.1.7 variant likely explains the increase of the pCFR during January and March that parallels the rise of the pCFR in Britain. This behavior offers further evidence that the B.1.1.7 VoC is more virulent than the original wild strain of SARS-CoV-2. Probably due to the excellent preparations regarding triage protocols taken by the German health care system, in mid-March 2021, the pCFR began to decrease toward its pre–B.1.1.7 level. Yet as testing and vaccinations for COVID-19 in Germany [15] lagged well behind the levels in the United Kingdom [19], reaching 10% full vaccination only in early May 2021, the pCFR increased by the end of May, most likely due to the more virulent B.1.617.2 (Delta) variant. That behavior is similar to that seen in the United States (Figure 2). By the beginning of July, full vaccination in Germany had reached 40%, and the pCFR showed signs of lessening to approximately 3%. The manifest periodicity in the number of daily deaths displayed in Figure 4 is due to the suppressed reporting of COVID-19 statistics on the weekends and justifies the use of smoothed sets of underlying data in computing the pCFR.

Doubtless, the variations in the national pCFR are also affected by the pervasiveness of national vaccination programs. To elucidate that influence, one can examine the variation in the pCFR versus the percentage of the total population who have been fully vaccinated (not including boosters). As booster programs become prevalent, plots of the case rate and pCFR versus percentage of boosted populations can also be revealing. An example related to the B.1.1.529 variant is offered in Multimedia Appendix 1, Section D.

### Effects of Comorbidities

An objection to relying on the initial analysis of the previous subsection is that the pandemic averaged case-fatality ratios remain dominated by the very high mortalities at the outset of the pandemic before appropriate and adequate isolation of the infected and modalities of treatment were understood. To mitigate that objection, one focuses only on the period of January 2021 through November 2021 that has been dominated by surges of VoCs that were described at the time of the WHO’s designation to have higher transmissibility and perhaps higher virulence than the original wild strain of SARS-CoV-2. In addition to accounting for the variations in the virulence and transmissibility of the new VoCs, one should also ask whether those variants exhibit significantly different sensitivity to physiological, environmental, and economic cofactors than were previously reported by Barletta [14].

**Figure 5:**
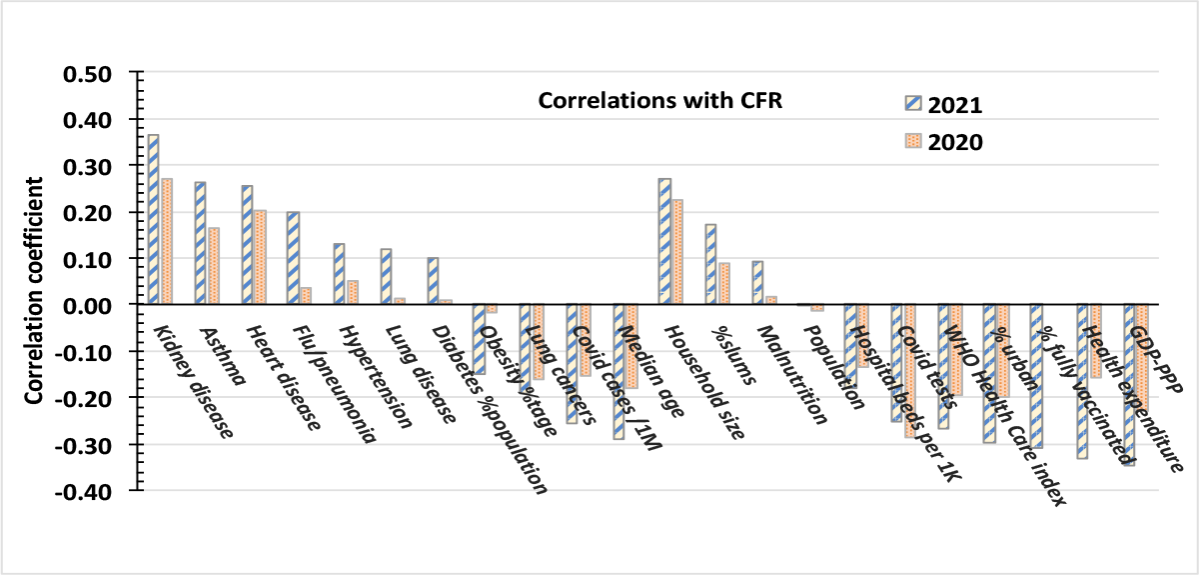
Linear correlations with the pandemic average CFRs in 2021, a variant-dominated period (striped), compared with values of the pandemic's averaged CFRs for 2020 (speckled). CFR: case-fatality rate; GDP-PPP: gross domestic product based on purchasing power parity; WHO: World Health Organization.

The SARS-CoV-2 statistics for 2021, correlated with the disease data of the European Renal Association–European Dialysis and Transplant Association (ERA-EDTA) Council [20] yield Figure 5. The striped bars account for the correlations only during the variant-dominated period of 2021. The speckled bars display the correlations of the pandemic averaged CFR throughout the pandemic dominated by the wild strain through December 30, 2020. With one exception, one sees no great differences between the variant-dominated and the pandemic-averaged values of 2020 beyond the general strengthening of previously observed trends. The tripling of the correlation between the average CFR with the total number of COVID-19 deaths per capita is likely due to deaths in unvaccinated populations caused by the B.1.617.2 (Delta) variant, which by September 2021 accounted for 80% of infections in the United States, according to the US CDC [21].

Apart from participants who participated in trials of the COVID-19 vaccines, no nation had begun a program of systematic immunization of its population in 2020. Therefore, the correlation value equals 0.0 for the vaccination cofactor in 2020 as shown in Figure 5.

Unlike a typical epidemiological analysis that would use the biological age of patients in a study, in this ecological data set, we must characterize the age distribution of an entire country by a single number. One might choose the median age of its population, the percentage of the population older than 65 years, or the life expectancy. As expected, these three characteristics are highly correlated, with correlation coefficients of 0.88 and 0.80 between the median age and the percentage older than 65 years and the life expectancy, respectively. However one characterizes age in a country, that value reflects social and economic aspects distinct from the physiological age of individual persons.

A detailed multivariate regression analysis of worldwide data including 24 independent variables reveals no constellation of cofactors, including the number of hospital beds per capita, that drives average national CFRs. The best regression model included only chronic kidney disease and the adjusted GDP as the independent variables. The *P* values for these variables were *P*=.01 and *P*=.02, respectively. The result for chronic kidney disease is consistent with findings of the ERA-EDTA Council [20]. That report indicates that, globally, the mortality risk from chronic kidney disease exceeds that from diabetes mellitus and chronic coronary disease, again in agreement with this analysis.

### Regional Variations

**Figure 6:**
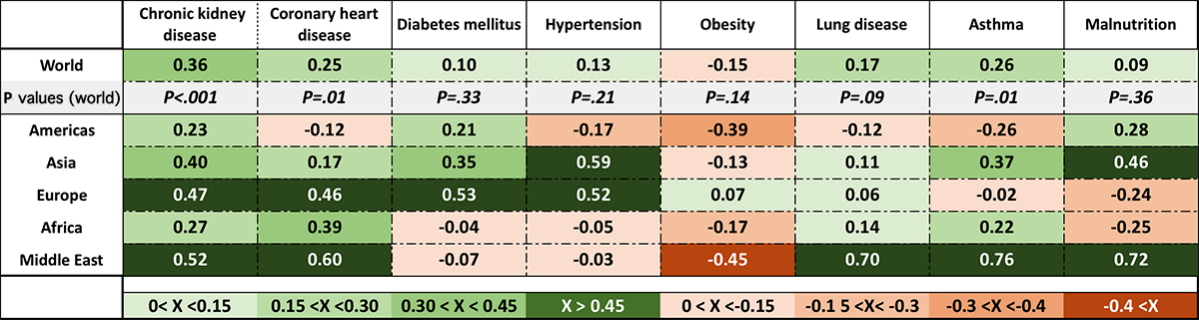
Heat map of regional variation of correlations with the case-fatality rate averaged over the period with *P* values for world data from January 2021 to December 2021.

A potential source of misinterpretation of the global statistics is the considerable variation of the correlations of cofactors from one region to another, as well as from the overall global values. Figure 6 compares the correlations of the pCFR for six commonly cited cofactors for the period dominated by the VoCs active during 2021. A restricted multivariate analysis over only countries in Europe reaches similar results.

The examination of statistics from Israel [22], which instituted an early and vigorous vaccination program early in 2021, could shed light on the role of testing and vaccination to suppress the serious consequences of infections with SARS-CoV-2. The smoothed data from OWID [15]—in Multimedia Appendix 1, Figure B.6—show evidence of an increase in the pCFR during mid-September 2021 consistent with an initial spread of the B1.617.2 variant in Israel. The spike in mid-May is likely spurious and too statistically insignificant due to the very low case rate to allow firm conclusions. Across all elements of the population of Israel, the overall vaccination rate is only 63%; however, the vaccination rate for persons ≥60 years of age is 80%. The variations in the pCFR from September through December are the result of the waning of the effectiveness of initial vaccinations [23], the rapid program of booster vaccination, and the increased virulence of the B1.617.2 variant.

**Figure 7:**
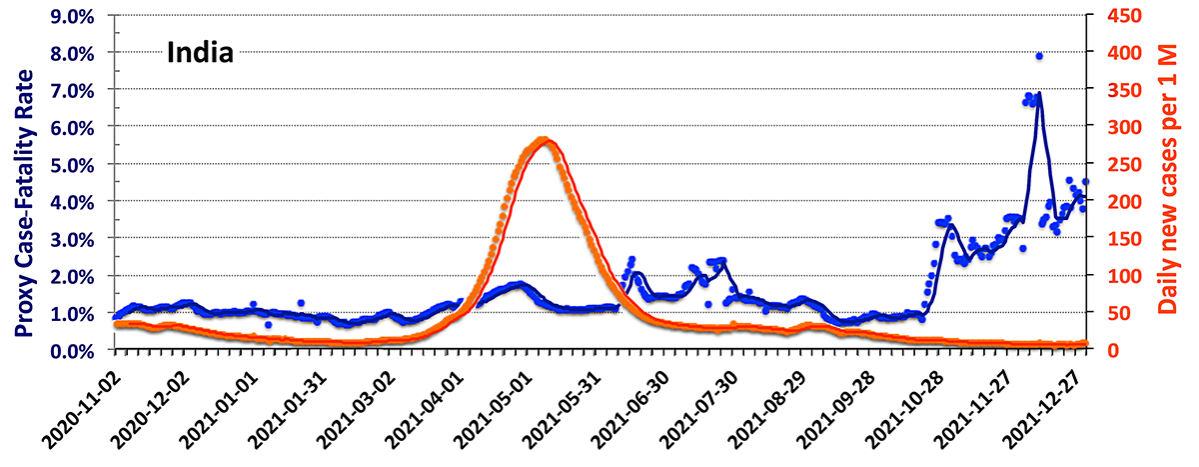
The smoothed values of the proxy case-fatality rate (blue) and daily new cases per 1 million persons (red) in India since November 1, 2020. The dark lines are the 7-day rolling averages.

Examining smoothed data [15] from India in a manner similar to that used to generate Figures 2-4 provides a useful day-by-day comparison among the countries. As was the case in the United States in July 2021, the dominant variant in India is B.1.617.2 (Delta), which started to become common in March 2021 [23]. Although the Indian data have less statistical noise than that from Israel, the smoothed day-by-day statistics of Figure 7 allow for a clearer look at temporal trends than do the raw data. Consistent with the increasing pervasiveness of the B.1.617.2 variant, the pCFR increases significantly from its February low of less than 1%, rising in March and April to 1.5% at the peak of the infection wave and to 2% by June when the surge was waning.

The results of Figure 7 are unlikely to include any significant effect of India’s program of vaccination that uses five different vaccines. By late-November 2021, only 30% of its population had been fully vaccinated [24]. Moreover, all the vaccines evaluated against B.1.617.2 appear to be roughly 10% less effective in controlling the development of COVID-19 in patients with the B.1.617.2 variant [25] than against the wild strain (at the 95% confidence level). The jump in the pCFR, seen in November 2021, occurred while the percentage of fully vaccinated persons was only 32% and the number of daily new cases remained at less than 6.3 per 1 million persons. During that entire period, the reproduction rate of the virus remained at less than 0.95.

**Figure 8:**
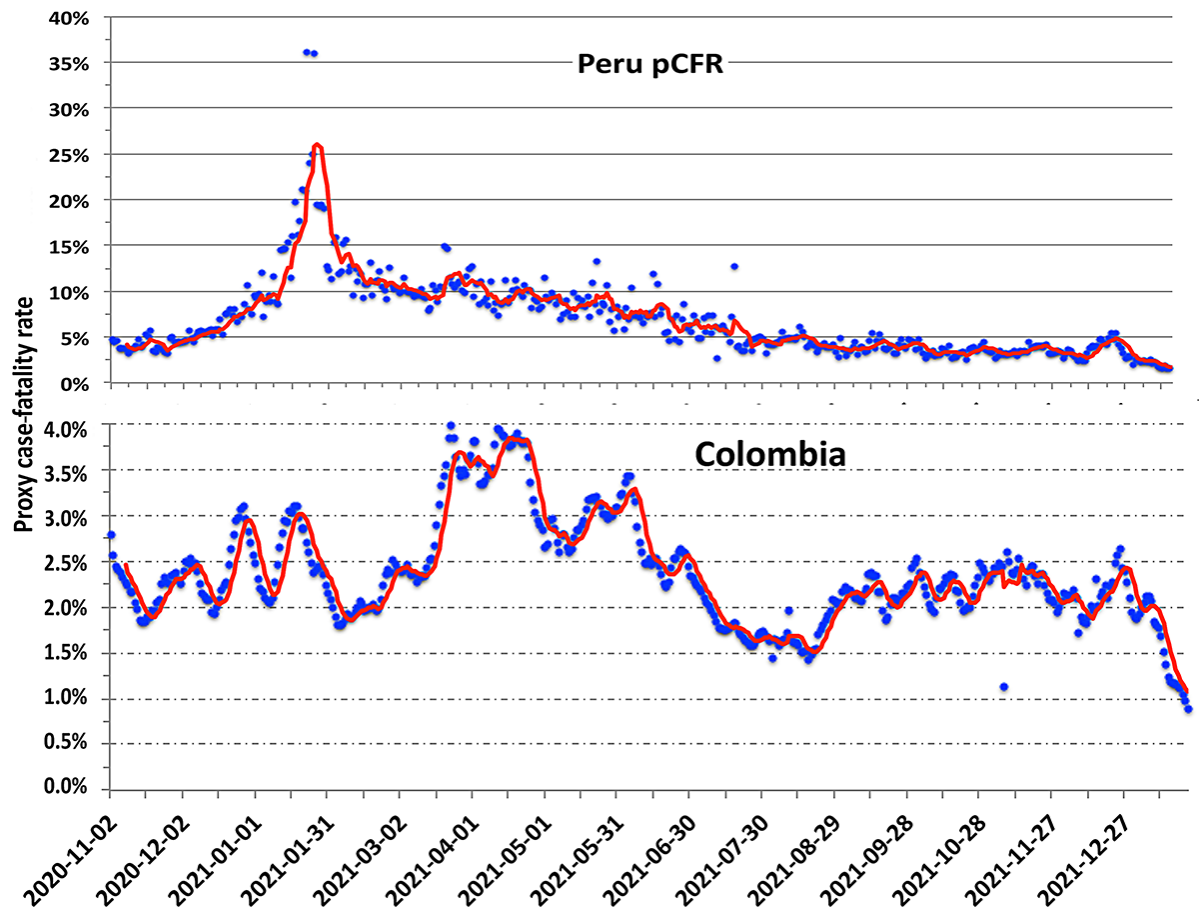
The behavior of the pCFR in Peru (upper plot) and Colombia (lower plot) since November 2020 based on the smoothed data of [15]. The red lines are the 7-day rolling averages. pCFR: proxy case-fatality rate.

The WHO-designated variant of interest, C.37 (Lambda), has been circulating widely in South America, having first been reported in Lima, Peru in December 2020 [26-28]. As shown in Figure 8, Peru saw a strong spike in the pCFR in January and February 2021, reaching 20%. Since that time, the pCFR has decreased gradually to roughly 5%. By July 2021, only slightly more than 10% of the Peruvian population had been fully vaccinated [15]; by November 2021, that number had increased to 49.4%.

The analysis of the C.37 virus by Kimura et al [27] identified a modified structure in the receptor binding domain of the spike protein that accounts for Lambda’s higher resistance to vaccine-induced immunity than is the case for the original wild variant. Hence, the initiation of the vaccination program in Peru cannot by itself account for the continuing decline in the pCFR. The South American scene is further complicated by the simultaneous circulation of multiple VoCs, particularly in Brazil, where the P.1 (Gamma) variant appeared in early 2021 [4,5].

**Figure 9:**
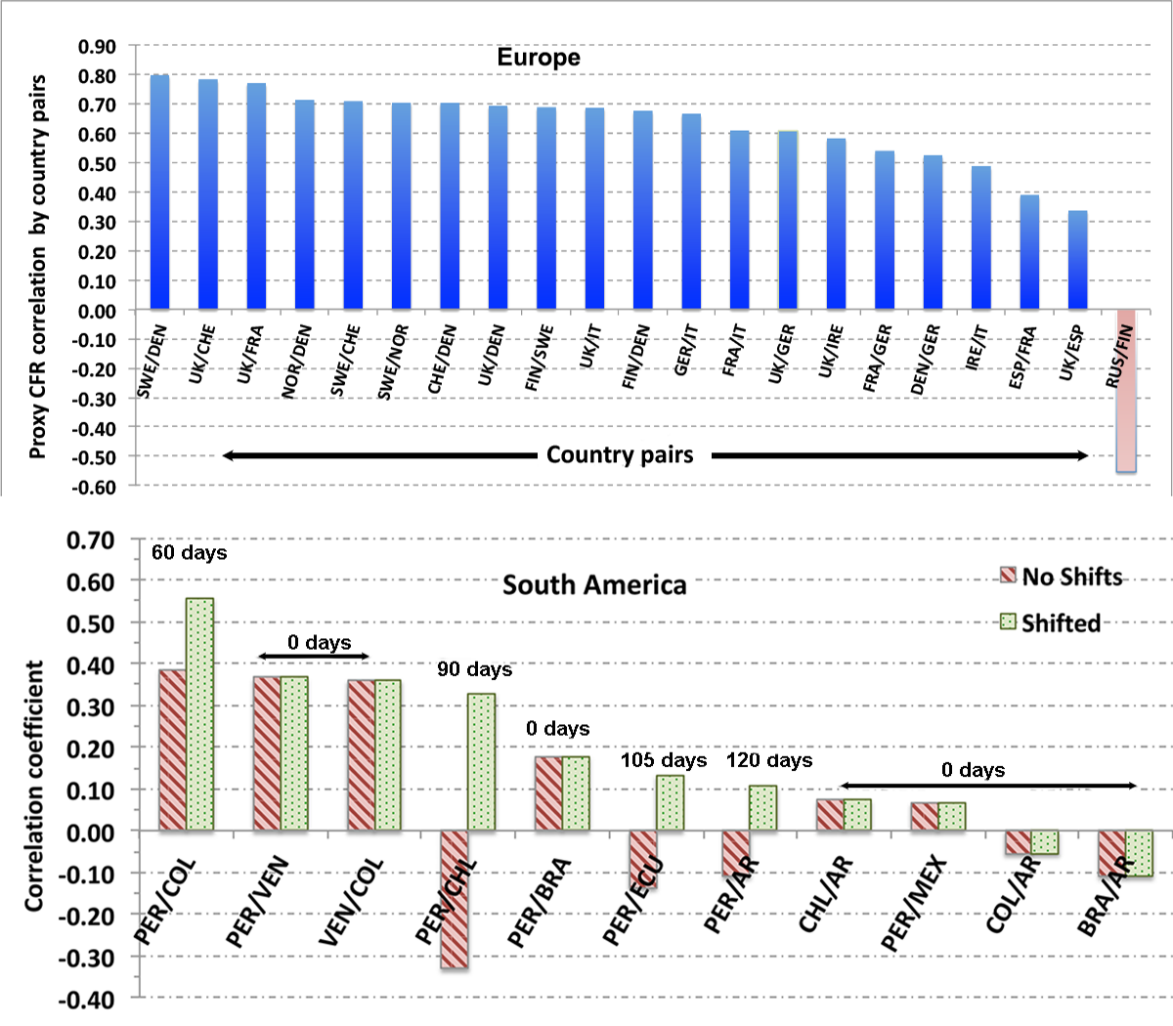
Correlations of proxy CFR between pairs of countries in Europe (top panel) and South America (bottom panel). CFR: case-fatality rate.

The lower panel of Figure 8 provides an example for Colombia. Comparison between plots of the temporal behavior of the pCFR (Figures 3 and 4 and Figures 7-9) can be made qualitative by computing the correlation *r* value for pairs of countries grouped into regions. One such set of calculations is displayed in Figure 9 for Europe and South America. The uncertainty in the correlation values is approximately ±0.05.

The upper panel of Figure 9 displays a strong to moderate correlation between countries in the Schengen region for which travel was relatively unhindered during the spread of the B.1.1.7 variant from the United Kingdom. Likewise, the country pairs in the Middle East—Iraq/Turkey, Turkey/Iran, and Iran/Iraq—show strong effects of transnational traffic during the Syrian civil war with correlations in the pCFR of 0.818, 0.711, and 0.634, respectively. Negative correlations in Figure 9 indicate either different courses of infection, treatment modality, vaccination program between the country pairs, or other impediments to the spread of a more infectious strain from one country to the other. An example of such a negative correlation, produced by neglecting the time delay in the spread of SARS-CoV-2 variants between countries is given in Multimedia Appendix 1 Section B, Figure B.7, which compares the time variation of the pCFR in Peru and in Argentina.

The distributions, when both are reckoned from November 2020, show no indication of the Lambda variant spreading from Peru to and through Argentina. One also sees no evidence of the Brazilian Gamma (P.1) variant causing a spike in the pCFR in Argentina (days 150-180 in Figure B.7) when the fraction of Gamma (P.1) cases was highest there. Even evidence of the spread of Lambda to Peru’s neighboring countries of Colombia and Venezuela, as reflected in the pCFR, are moderate-low, with *r* values of 0.38 and 0.37, respectively. In fact, the values for Chile and Argentina are negative, –0.33 and –0.11, respectively. However, shifting the Peruvian distribution later in time (ie, day 1 for Peru corresponds to Day 1 + X_delay_) to account for the time of the variant to propagate from one country to another increases the correlation coefficients significantly.

The striped bar in the PER/COL pairing in Figure 9 shows that shifting the Peruvian profile 60 days later in time, introduces a much larger similarity with the Colombian profile of the pCFR with the *r* value of 0.56 (speckled bar). Moreover, the correlation in the distribution of new cases shifts to 0.68. For Argentina, the correlation in the pCFR increases to 0.11 for a 120-day shift, while the correlation of the distribution of new cases increases to 0.86.

Such a large value of X_delay_ is consistent with the Delta variant being first reported in Argentina in August 2021 [29]. The differences in the pCFR in new cases in Argentina could be caused by differences in effective treatment in the two countries or by differences in the predominant variants of SARS-CoV-2. Notably, Peru displays no evidence of a peak in the pCFR due to the B.1.617.2 in August when the fraction of Delta cases peaked [30].

The light purple bar in the PER/COL pairing in Figure 9 shows that shifting the Peruvian profile 60 days later in time introduces a much larger similarity with the Colombian profile. Additionally, the correlation of new cases shifts to 0.68. One may interpret these results as indicating the amount of time needed for the Lambda variant to spread widely into Colombia, where its prevalence is now high [31]. Further obscuring the degree to which the Lambda variant has spread out of Peru has been the competition in Peru between the Lambda variant and the Gamma variant. That competition has been examined by Vargas-Herrera et al [30].

For North America, one observes only a moderate correlation in the pCFR (0.57) between the United States and Canada. That low value may be explained by differences in the US and Canadian health care systems and by the fact that the border between the countries was closed since March 2020 through most of 2021.

### Effects of COVID-19 Vaccines

As COVID-19 vaccines have been broadly reported by Barda et al [32] to be effective in reducing the severity of infections that nevertheless occur, one must account for a vaccine effect when using a metric based on CFRs. This study uses plots of the pCFR versus the percentage of population fully vaccinated and versus the percentage of population receiving a booster shot to discern waves of infection due to different variants. Examples relevant to the B.1.1.529 VoC are given in Multimedia Appendix 1 (Figure D.1, D.2, and D.3) that display the simultaneous spike in infection accompanied by a strong reduction in the pCFR. In comparing countries with different vaccination profiles, time series measured in days from November 2020 were used.

## Discussion

### Summary of Findings

#### Finding for Objective 1

The proxy for the daily CFR, pCFR, as defined by equation 1 and computed over a smoothed distribution of the deaths attributed to COVID-19 infections, provides a useful metric to track the national dynamics of the spread of SARS-CoV-2 VoCs overlaid with the implementation of that country’s vaccination program. The variations in risk of mortality, especially near the first appearance of new VoCs, is clearly seen in the time series of values of the pCFR.

The example of the United Kingdom is instructive in this regard. A clear increase in the fatality rate due to the increased virulence of the B.1.1.7 variant is followed by the sharp decrease in the daily CFR to about 0.25% thanks to the United Kingdom’s aggressive program of vaccination [18] as confirmed by the clinical study of Challen et al [33]. That low rate persisted despite the spread of the Delta variant throughout the United Kingdom. A similar increase in mortality due to B.1.1.7 was later observed in the pCFR data for the United States as displayed in Figure 2.

#### Finding for Objective 2

Using the pCFR, one finds that the influence of both economic and medical cofactors on the rate of fatalities due to infections caused by WHO-designated VoCs remains similar albeit somewhat strengthened with respect to the levels found for the wild strain of SARS-CoV-2. Based on a detailed global regression analysis, the strongest observed single correlation globally is 0.36 (SD 0.02), with *P*<.001 for chronic kidney disease for January through November 2021. No other physiological cofactors displayed positive linear global correlations, exceeding 0.26 for asthma with *P*=.008 and coronary heart disease with *P*=.01.

#### Finding for Objective 3

Strong regional variations of the influence of all categories of cofactors observed for the wild strain persist in the infections due to all VoCs. That variation emphasizes the effect on COVID-19 mortality due to regional differences in national economics, in patterns of national health policies, and possible variations in cultural and environmental factors. Moreover, the regional variations that appear in Figure 6 can explain some of the conflicting observations of risk factors found especially in the literature published or e-published in 2020.

### Limitations

A limitation of using the pCFR metric is that large fluctuations in the pCFR can occur when the daily caseload is low. Whether these fluctuations are driven by transmission among small clusters of individuals with similar medical conditions or in small communities without adequate medical facilities cannot be discerned without detailed patient data. Nationally aggregated public health data are not sufficient.

For most countries studied, the pCFR successfully tracks waves of reinfections as well as the introduction and propagation of new VoCs (Figure 2, Figure 5, Figure 7, and Figure 8). The timing of strong increases in the daily pCFR in the United States and Germany from June through late July 2021, peaking at 2.0% and 4.5%, respectively, correspond to the rapid spread of the B1.167.2 variant and support the characterization of Delta as being both more virulent and more contagious than the original wild strain. Despite the moderate success of its vaccination program, the pCFR in the United States continued to increase in August 2021. In contrast, the pCFR in Germany decreased by early August to a value of roughly 1%. By October 2021, the pCFR began to increase in both countries due to a resurgent B1.167.2 wave.

The prevalence of multiple coexisting conditions also varies from region to region, partially explaining the regional variations seen in Figure 6. If a generally accepted measure of the readiness of the immunity system to fight infection were available, as was proposed by Han [34], one might obtain a clearer definition of COVID-19 mortality risk factors. However, producing a large database of Han’s [34] metric would require genetic sequencing of large representative samples of individuals in a broad range of countries.

### Comparison With Prior Studies

The results for objective 2 are consistent with the findings of the ERA-EDTA Council [21] although inconsistent with the finding of the July 2020 literature review and meta-analysis of Singh et al [35] and Bajgain et al [36] that found diabetes and cardiovascular diseases as the most common cofactors. That is not to say that other cofactors may not be seen in many patients who die from COVID-19; the correlation coefficients for patients with coronary heart disease and diabetes mellitus are high enough that persons with those conditions should take extra prophylactic precautions against infections.

Following the method of Ranard et al [11] for a data set limited to November 2020 through November 2021, this study finds minimal quantitative differences with the conclusion of Ranard et al [11] that the most commonly cited comorbidities do not per se substantially increase the risk of serious consequences of SARS-CoV-2 infections. However, one cannot ignore that many persons with such conditions frequently have multiple cofactors and have either an inherent or medication-depressed level [37] of immune function that can worsen the effects of a COVID-19 infection.

Consistent with Solis-Moreira [32], the time series of the pCFR show only weak evidence for significant spread of the Lambda and Mu variants of interest outside of South America, although some cases have been seen in Europe and North America. However, careful examination of the correlation of the pCFR distributions shows delays consistent with the times that Lambda appeared in countries not having a common border with Peru or Colombia.

While the epidemiological data are still too early to draw conclusions with respect to the highly contagious Omicron (B.1.1.529) variant, its worldwide spread provides a testing ground for many of the ideas presented herein. The early spread of the variant is described in Multimedia Appendix 1, Section D. Already, limited statistics support the hypothesis that this VoC is more transmissible and significantly less virulent than the B1.167.2 variant. The degree to which booster vaccinations and strict prophylactic measures can suppress both severity and extent of this VOC requires further detailed analysis. Whether new mutations derived from Omicron (B.1.1.529) retain such properties in addition to vaccine evasion is a topic for future research.

### Conclusions

This research explores the degree to which SARS-CoV-2 VoCs generate waves of fluctuations in CFRs, increase the risks of mortality to persons with certain comorbidities, and respond to public health initiatives with decrease risks of mortality as the percentage of fully vaccinated populations increases. The pCFR that was introduced to address these issues is a valid proxy for national rates of the level of virulence of the VoCs. Waves of infection due to VoCs and their spread are generally, but not always, manifest in daily variations of the pCFR. An exception is the behavior of the pCFR (Figure 3) for B1.167.2 infections in the United Kingdom; however, in most cases, the temporal variations of the pCFR show strong correlations in propagation of VoCs. For example, the temporal distribution of the pCFR in Germany (Figure 4) shows distinct peaks coinciding with the spread of B1.1.7 and B1.167.2 throughout the Schengen zone.

This study tested the hypothesis that apparent increases in the virulence of VoCs might be due to increased susceptibility to severe infection in persons with certain comorbidities. The comparison of Figure 5 does not substantiate that hypothesis.

Robust programs of vaccination can alter dynamics of VoCs by lowering the pCFR averaged over monthlong periods as shown by the experience of the United Kingdom. However, complete suppression of the pCFR to uniform low levels is not always seen; such an example for Italy appears in Multimedia Appendix 1, Figure D.1. Plotting the pCFR, case rate, and reproduction number, R_o_, against the percentage of total population fully vaccinated allows one to account for the effect of vaccinations; however, large variations in the pCFR and R_o_ can persist in countries with vaccination rates >60% (Multimedia Appendix 1, Table D.1 and Figure D.2). As the efficacy of vaccines wanes after several months [38], one should also plot metrics with respect to the percentage of the populations receiving booster shots.

## Appendix

Multimedia Appendix 1 Supplementary tables and figures.

## Abbreviations

CDC: Centers for Disease Control and Prevention
CFR: case-fatality rate
ERA-EDTA: European Renal Association–European Dialysis and Transplant Association
GDP: gross domestic product
OWID: Our World in Data
pCFR: proxy case-fatality rate
VoC: variant of concern
WHO: World Health Organization
