# Supplementary material for: The Influence of SARS-CoV-2 Variants on National Case-Fatality Rates: Correlation and Validation Study
Source: JMIRx Med. 2022 May 24;3(2):e32935. doi: 10.2196/32935 (PMC9364421; doi:10.2196/32935)
Supplement: Multimedia Appendix 1 [file xmed_v3i2e32935_app1.docx]

# Appendix Added in Proof

to

**SARS-CoV-2 variants of concern:**

# Influences on national case fatality rates

Section A

Table A. The countries sampled are grouped into the same five regions as in reference [12].

| Region | Population (million) | Countries |
| --- | --- | --- |
| Americas | 977 | Argentina, Bolivia, Brazil, Canada, Chile, Columbia, Costa Rica, Dominican Republic, Ecuador, El Salvador, Guatemala, Honduras, Mexico, Panama, Paraguay, Peru, United States, Venezuela |
| Asia | 2504 | Australia, Bangladesh, China, India, Indonesia, Japan, Kazakhstan, Kyrgyzstan, Korea, Malaysia, Nepal, New Zealand, Pakistan, Philippines, Singapore, Thailand, Taiwan |
| Europe | 725 | Albania, Armenia, Austria, Azerbaijan, Belarus, Belgium, Bosnia, Bulgaria, Croatia, Czechia, Denmark, Estonia, Finland, France, Germany, Greece, Hungary, Ireland, Italy, Macedonia, Moldova, Netherlands, Norway, Poland, Portugal, Romania, Russia, Serbia, Spain, Sweden, Switzerland, Ukraine, United Kingdom |
| Africa | 768 | Algeria, Cameroon, Congo, Ethiopia, Ghana, Ivory Coast, Kenya, Libya, Madagascar, Mali, Morocco, Nigeria, South Africa, Sudan, Uganda, Zambia |
| Middle East | 487 | Afghanistan, Bahrain, Egypt, Iran, Iraq, Israel, Lebanon, Kuwait, Oman, Qatar, Saudi Arabia, Turkey, United Arab Emirates, Uzbekistan, Yemen |

# The analysis of this study uses the standard definition of the Pearson linear correlation factor as given in Equation A.1.

#

# Multivariate regression analysis of this study uses the standard Data Analysis package of Excel version 16.43.

Section B

This section shows an analysis of two alternate modes of examining the time series of case fatality rates. As both suggested modes are differential metrics, the level of noisy is much higher than in either the raw data or the integrative 7-day rolling average approach.

#

# Figure B.1 The derivative measure R14(N) for the United Kingdom. A rolling 7-day average (red curve) is applied to the time series.

# In contrast the rolling 7-day average (red curve) of pCFR of Figure B.2 shows features lost in the differential metric. Moreover, the ragged features in R14 from days 149 to 240 occur when the cases load and the number of cases required ICU admission were low [12].

#

# Figure B.2 A sample rolling 7-day average (red curve) of pCFR for the United Kingdom.

# The differential metrics described in the main text are permeated by high frequency noise as can be seen from a Fourier analysis of pCFR and the day-to-day ratio in Figure B.3.

#
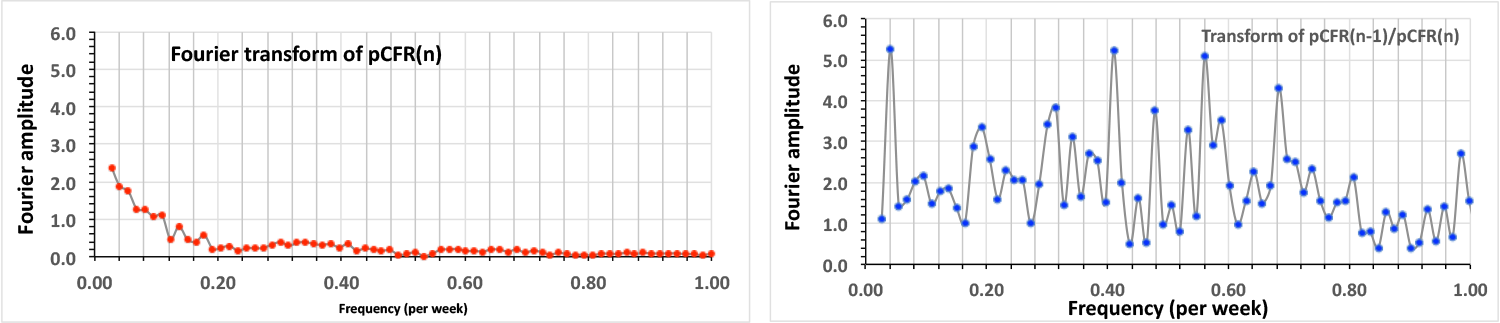


# Figure B.3 Comparison of fast Fourier transforms of the time series of pCFR (left) and of the transform of the day-to-day ratio of pCFR(right).

#
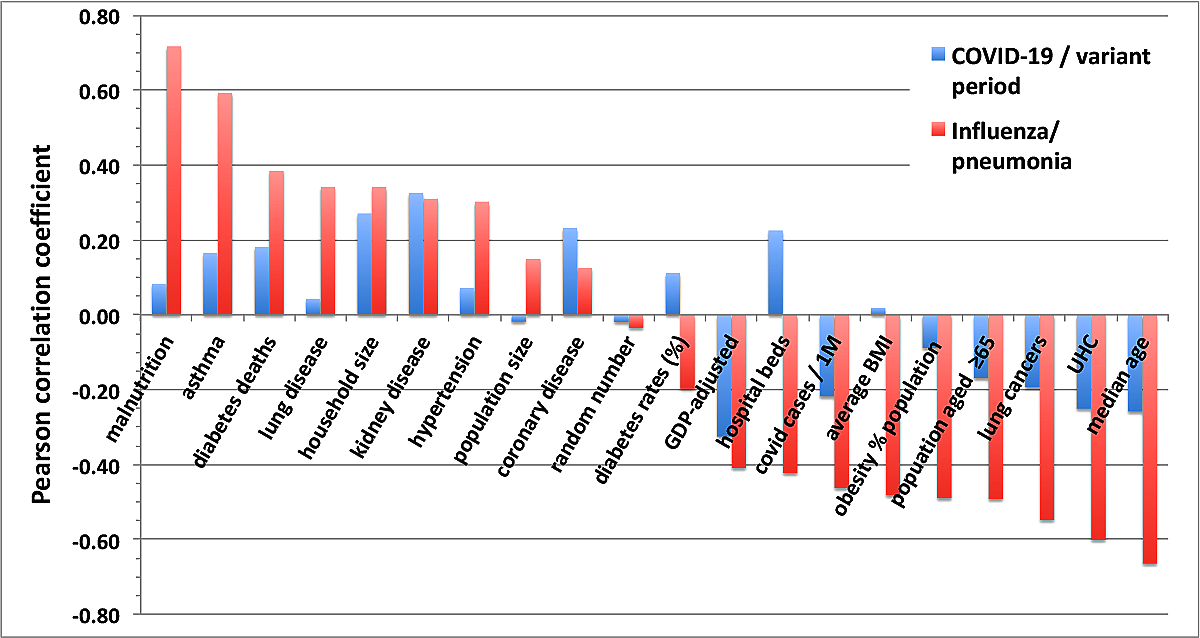


# Figure B.4 Comparison of covariate influences on COVID-19 fatality during 2021 and on outcomes of influenza initiated pneumonia illustrates how different SARS-CoV-2 is from influenza.

# One may question whether regional differences in disease outcome are due to differences in the number of hospital beds or ICU units available in a country. Not all countries make ICU data readily available, but a look at the correlation of the number of hospital facilities indicates no obvious correlations

#
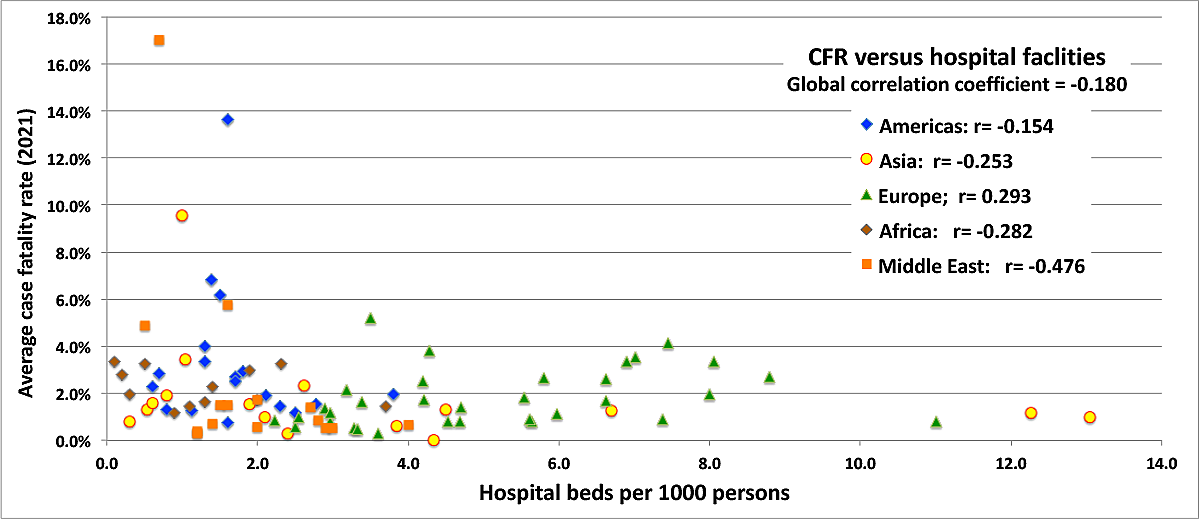


# Figure B5. An example of the average CFR versus the number of hospital beds per 1 million persons broken down by region. One sees little evidence of correlation.

# An example of some of the effects of robust vaccination programs can be seen in the case of Israel, which began vaccinations in early January 2021. Vaccinations led to a steady decrease in the infection rate avoiding any surge due to the B1.1.7 variant. That decrease was accompanied by a decrease in pCFR. As the vaccination rate (of total population) surpassed 50%, the infection rate dropped sharply. One sees a limitation of the using pCFR as a sole metric. When infection rates are very low, small clusters of severe cases can manifest as large variations in pCFR.

# As vaccine efficacy began to wane for a large fraction of the population the case rate began an increase roughly overlapping the arrival of the B1.617.2 variant and the onset of a vigor program of vaccine booster shots


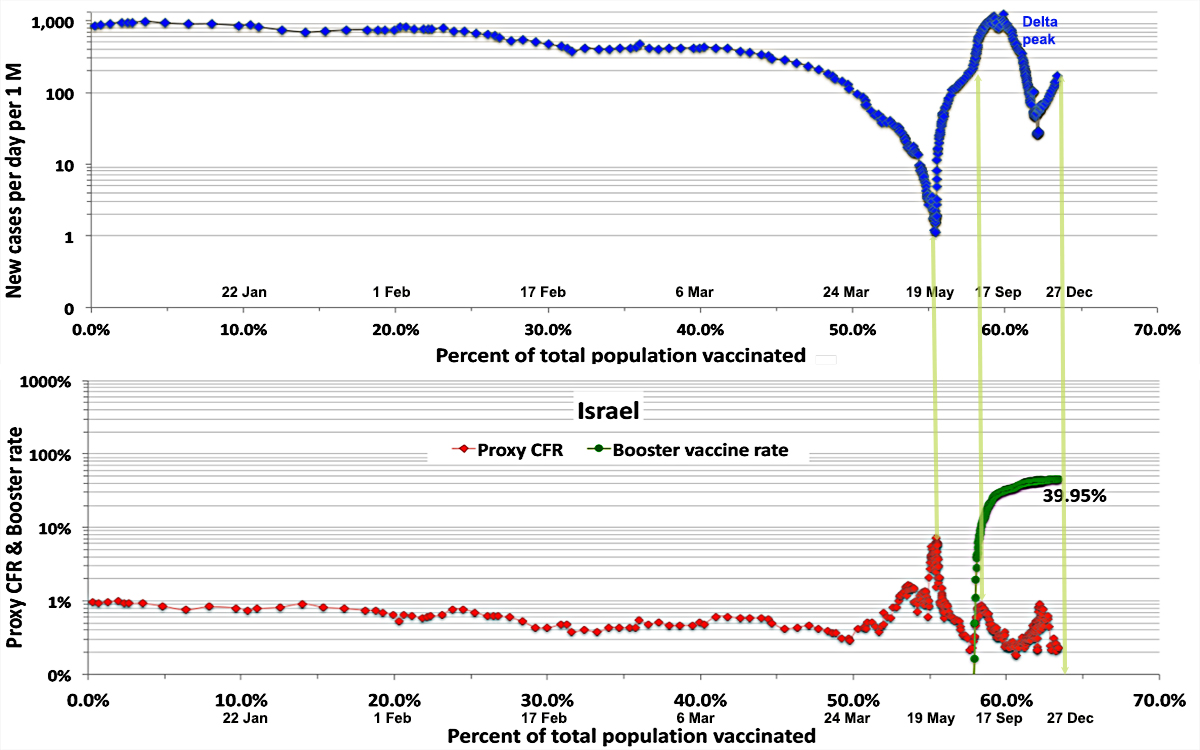


Figure B.6. Smoothed daily “CFR” in Israel from November 1, 2020. The effects of vaccines on reducing fatality rate are apparent both prior to and during the wave of B1.617.2 infections


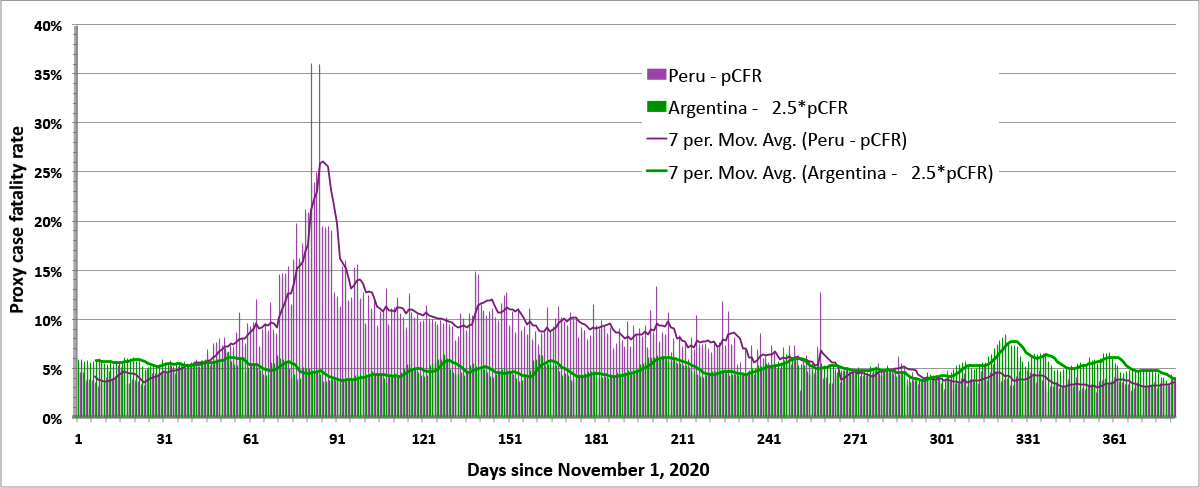


Figure B.7. Example of a country pair exhibiting a significant negative correlation due to neglecting the time delay in spread of SARS-CoV-2 variants between Peru and Argentina.

# Section C: Plots of pCFR versus potential co-factors.

# The first phase of the correlation analysis was to plot infection rates and case fatality rates against all potential covariates that were considered to look for manifest non-linear effects. In all cases the countries were specified by their respective regions in the graphs. This section shows several representative examples of the initial set of plots. As was indicated in Figure 5 of the main text. The next step was to search for correlations across the global set of countries as well as with countries disaggregated by region. The final figure of this section displays a heatmap of the cross-correlations among all covariates and their correlation with the average CFR for the 2021, when variants of concern dominated infections globally.

#
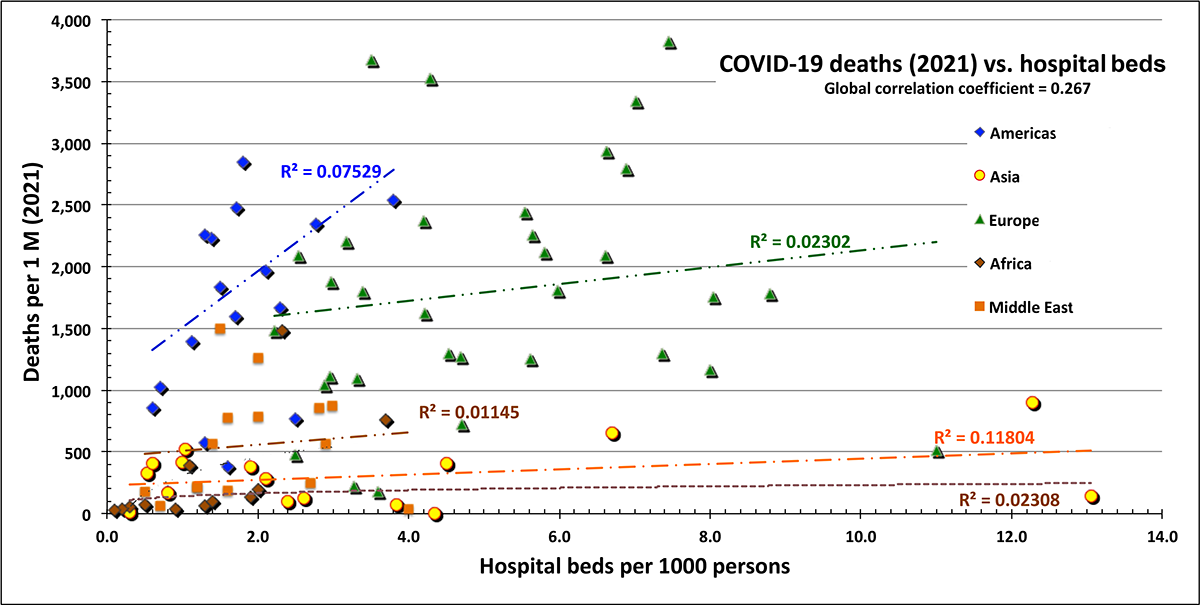


# Figure C.1 The plot of deaths per 1 million versus hospital facilities does not sustain the conjecture that increases in death rates are due, in general, to a lack of hospital facilities. Logarithmic trend lines for the separate regions are added to guide the eye

#
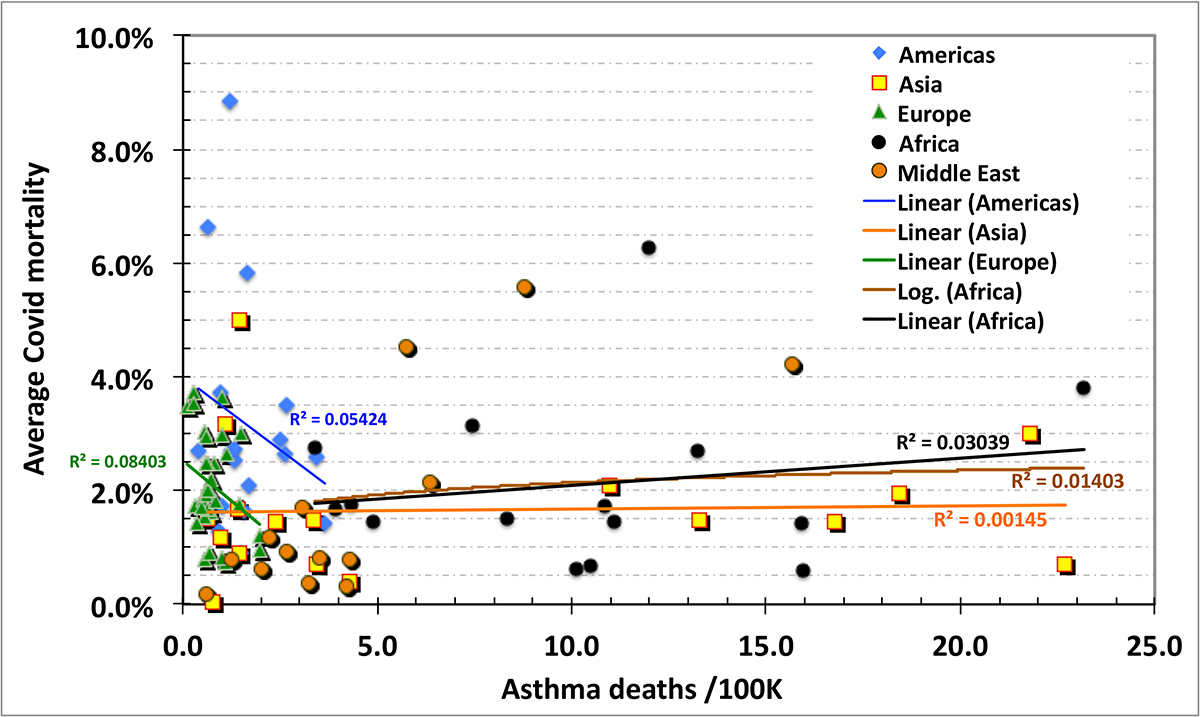


# Figure C.2 Little correlation of COVID-19 case fatality rate with incidence of severe asthma is seen except in the Middle East, either globally or on a regional basis

#
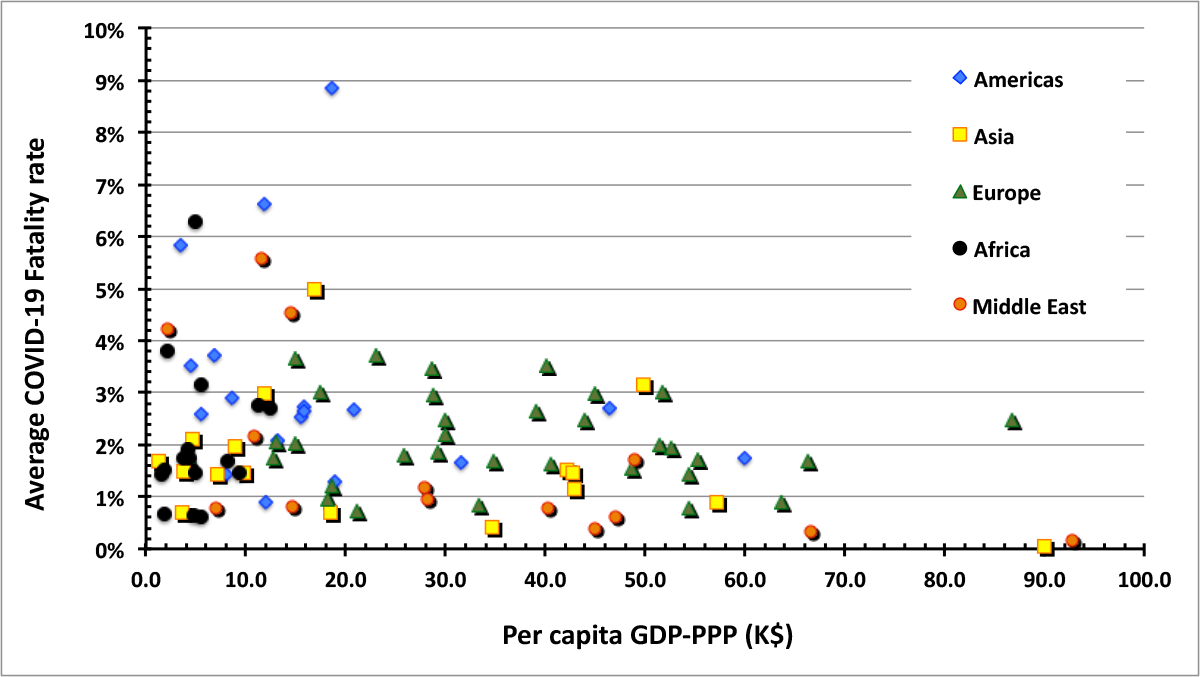


# Figure C.3 Any dependence of COVID-19 mortality on adjusted gross domestic product per capita displays strong regional variations

#
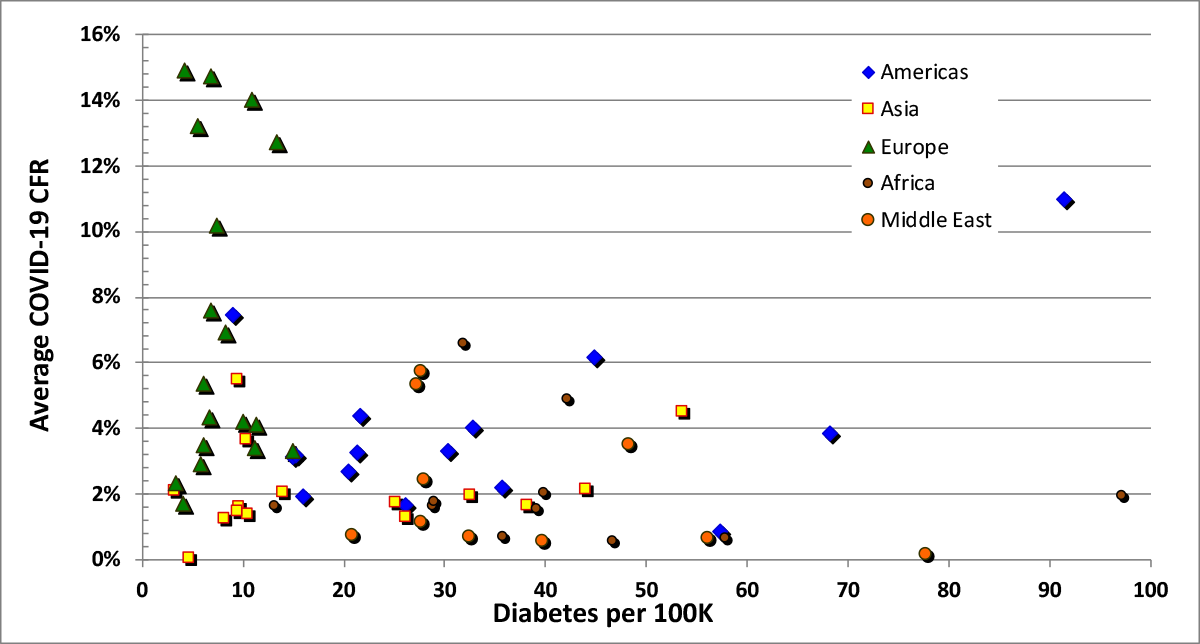


# Figure C.4a Any dependence of COVID-19 mortality on incidence of diabetes mellitus has a strong regional variation

#
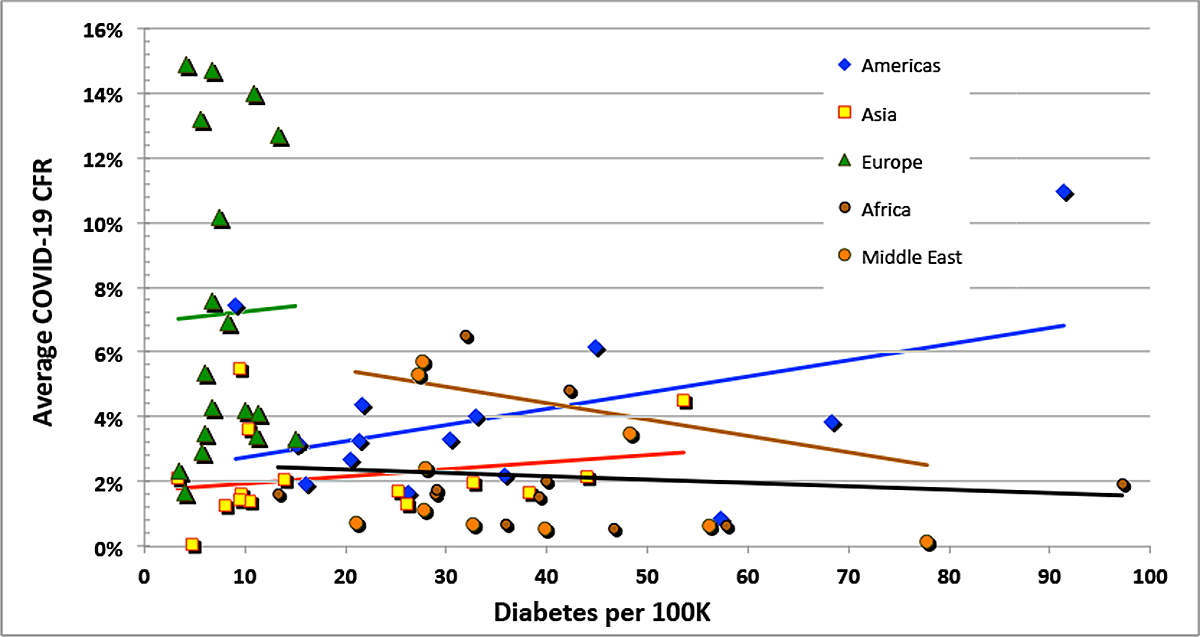


# Figure C.4b Trendlines for each region do not support the assumption of strong non-linearities in dependence of COVID-19 on the incidence of diabetes mellitus

#
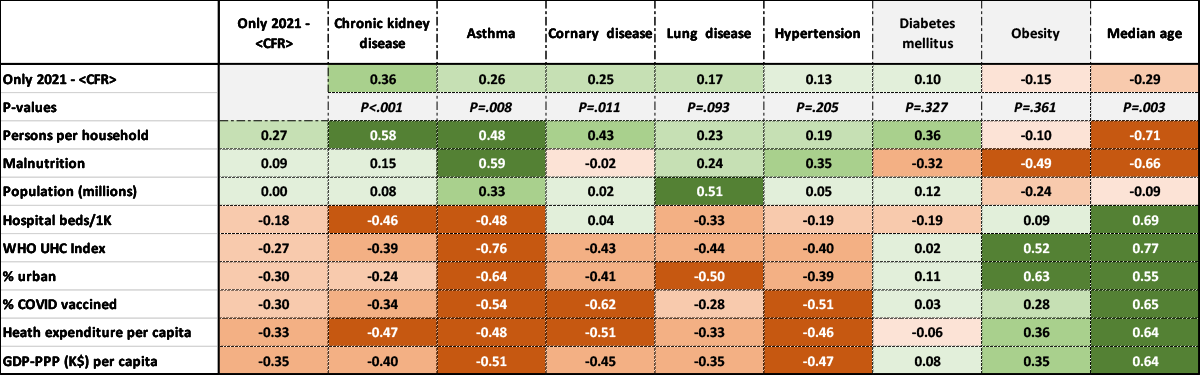


# Figure C.5. Heatmap of global correlations among independent variables and with CFR.

# Section D: The Omicron (B.1.1.529) Variant

# The Omicron variant from South America began its spread near the end of the present study. Limited epidemiological statistics support the hypothesis that this VoC is more transmissible and less virulent than the B1.167.2 variant. The degree to which booster vaccinations and strict prophylactic measures can suppress both severity and extent of this VOC requires detailed analysis. An example for Italy, shown in Figure D.1, illustrates the characteristics the behavior of a extremely contagious less virulent VoC: that is, a pronounced decline in pCFR and an accompanying very rapid increase in the number of cases despite a high rate of vacations in the population.

#
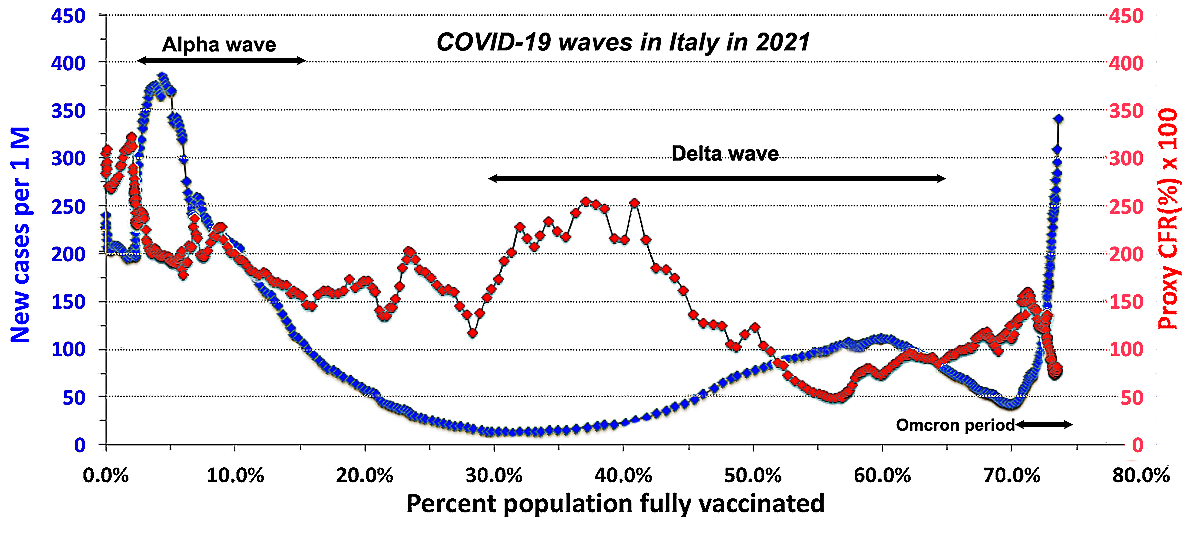


# Figure D.1 The variation of rate of infection (blue) and CFR (red) on the degree of vaccination in Italy through the Alpha, Delta, and beginning of the Omicron wave. The curves in the Omicron period suggest the increase transmissivity and lower virulence of that variant.

# Though not as dramatic as the Italian example, similar behavior is suggested by the evolution of PCFR and the reproduction number, R_o_, listed in Table D.1 for a range of countries considered in the present study.

# Table D.1 The evolution of the SARS-CoV-2 reproduction rate early in the wave of the Omicron VoC.

| **Country** | **Lowest Ro** | **% fully vaccinated Dec. 15** | **Ro - Nov. 1** | **Ro - Dec.15** | **pCFR (%) - Nov. 1** | **pCFR (%) - Dec. 15** |
| --- | --- | --- | --- | --- | --- | --- |
| **Austria** | 0.58 | 69.3% | 1.43 | 0.61 | 0.71% | 0.47% |
| **Belgium** | 0.66 | 75.4% | 1.47 | 0.80 | 0.47% | 0.25% |
| **Bulgaria** | 0.61 | 27.9% | 1.05 | 0.94 | 4.67% | 3.89% |
| **Czech Republic** | 0.61 | 60.9% | 1.54 | 0.82 | 1.56% | 0.61% |
| **Denmark** | 0.57 | 77.4% | 1.44 | 1.25 | 0.20% | 0.12% |
| **Estonia** | 0.61 | 60.9% | 1.09 | 1.04 | 0.89% | 0.84% |
| **Finland** | 0.93 | 73.6% | 1.18 | 1.23 | 0.45% | 0.50% |
| **France** | 0.66 | 71.6% | 1.02 | 1.18 | 0.61% | 0.39% |
| **Germany** | 0.53 | 69.3% | 1.05 | 1.03 | 0.71% | 0.66% |
| **Greece** | 0.76 | 65.8% | 1.25 | 0.98 | 1.72% | 1.50% |
| **Hungary** | 0.54 | 61.5% | 1.69 | 0.77 | 4.53% | 1.77% |
| **Ireland** | 0.92 | 76.6% | 1.12 | 0.97 | 0.66% | 0.15% |
| **Italy** | 0.56 | 73.5% | 0.92 | 1.10 | 1.17% | 0.81% |
| **Netherlands** | 0.63 | 71.0% | 1.33 | 0.81 | 0.42% | 0.28% |
| **Norway** | 0.74 | 71.2% | 1.35 | 1.33 | 0.24% | 0.31% |
| **Poland** | 0.43 | 55.0% | 1.41 | 0.89 | 3.17% | 1.79% |
| **Portugal** | 0.49 | 88.9% | 1.12 | 1.18 | 0.54% | 0.51% |
| **Russia** | 0.94 | 42.5% | 1.12 | 0.94 | 4.51% | 3.40% |
| **Spain** | 0.70 | 80.8% | 1.10 | 1.62 | 2.19% | 0.51% |
| **Sweden** | 0.46 | 72.0% | 1.28 | 1.50 | 7.40% | 0.31% |
| **Switzerland** | 0.61 | 66.3% | 0.87 | 1.28 | 0.29% | 0.30% |
| **United Kingdom** | 0.69 | 68.7% | 1.17 | 1.08 | 0.35% | 0.26% |
| **Iran** | 0.75 | 60.2% | 0.93 | 0.78 | 1.36% | 1.51% |
| **Iraq** | 0.71 | 12.7% | 0.83 | 0.71 | 1.65% | 1.67% |
| **Israel** | 0.46 | 65.0% | 0.61 | 1.14 | 0.37% | 0.42% |
| **U.A.E.** | 0.63 | 90.3% | 0.83 | 1.08 | 0.56% | 0.41% |
| **Nigeria** | 0.67 | 2.0% | 0.83 | 1.71 | 0.99% | 0.51% |
| **South Africa** | 0.56 | 25.6% | 0.72 | 1.26 | 4.92% | 0.92% |
| **Australia** | 0.69 | 75.3% | 0.82 | 1.55 | 0.65% | 0.45% |
| **India** | 0.68 | 38.3% | 0.88 | 0.91 | 3.51% | 3.84% |
| **Japan** | 0.41 | 77.9% | 0.67 | 1.13 | 1.88% | 0.89% |
| **Pakistan** | 0.74 | 26.0% | 0.84 | 0.97 | 1.23% | 1.91% |
| **Singapore** | 0.62 | 87.0% | 0.96 | 0.57 | 0.43% | 0.36% |
| **S. Korea** | 0.64 | 81.7% | 1.21 | 1.13 | 0.83% | 1.54% |
| **Taiwan** | 0.61 | 63.9% | 1.00 | 1.14 | 2.33% | 1.54% |
| **Argentina** | 0.69 | 69.0% | 1.09 | 1.21 | 1.92% | 1.18% |
| **Bolivia** | 0.87 | 37.4% | 1.17 | 1.16 | 1.04% | 1.33% |
| **Brazil** | 0.90 | 66.0% | 0.97 | 0.85 | 3.13% | 1.28% |
| **Canada** | 0.68 | 76.9% | 0.85 | 1.09 | 0.98% | 0.07% |
| **Chile** | 0.70 | 85.2% | 1.28 | 0.70 | 1.29% | 1.44% |
| **Colombia** | 0.65 | 51.8% | 0.96 | 0.84 | 2.00% | 2.01% |
| **Ecuador** | 0.66 | 68.0% | 0.66 | 0.93 | 0.51% | 3.12% |
| **Peru** | 0.93 | 60.2% | 1.02 | 1.09 | 3.61% | 4.80% |
| **United States** | 0.72 | 60.6% | 0.90 | 1.17 | 1.58% | 1.27% |

# Systematic effects of strong vaccination programs are suggested in Figure D.2 which separates European countries, which have been especially hard hit by Omicron, from the rest of the world in which Omicron has been less prevalent by mid-January 2022. Figure D.3 for Denmark shows that even very high vaccination rates accompanied by intensive booster shot administration has not appreciable showed the wave of Omicron infections.

#
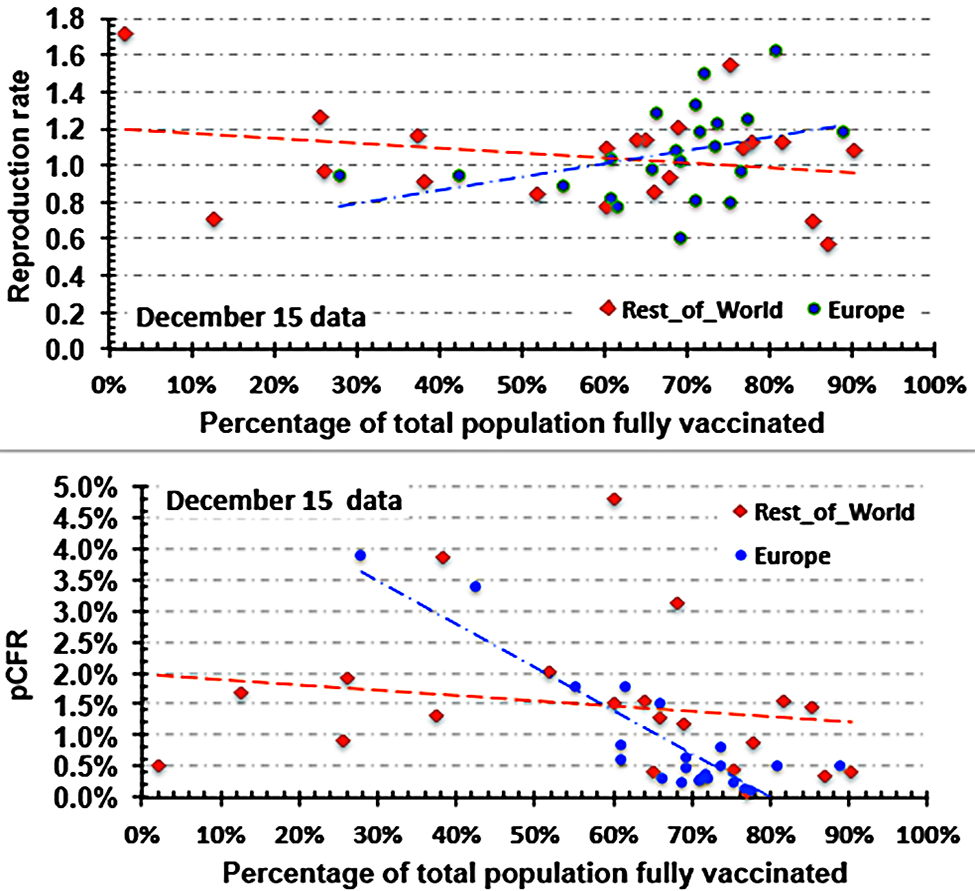


# Figure D.2 Effects of increased vaccination rates on viral reproduction number and pCFR through the beginning of the Omicron wave. Linear trendlines, matching the color of the data points from Table D.1, suggest the increase transmissivity and lower virulence of the Omicron variant.

#
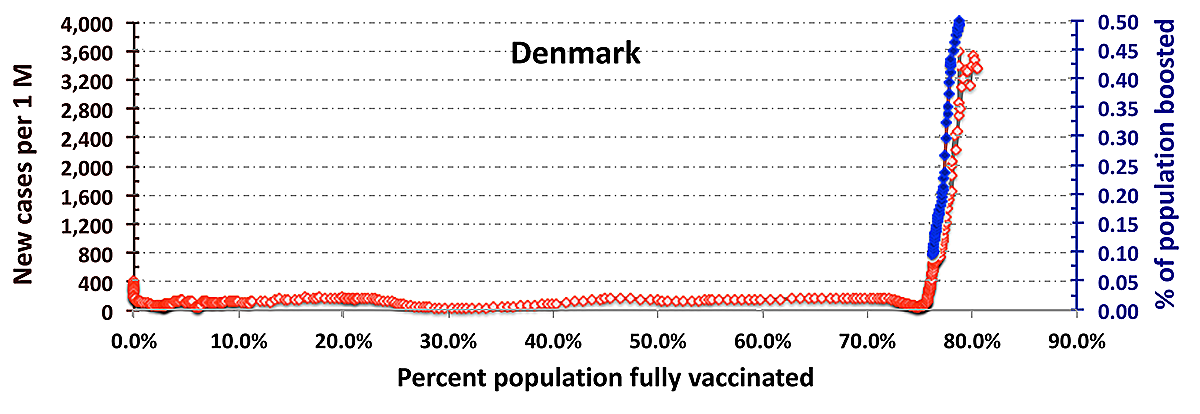


# Figure D.3 The variation of rate of infection (open markers) on the degree of vaccination and boosters (solid markers) in Denmark through the Delta, and beginning of the Omicron waves
